# Safety and Performance of the Tandem t:slim X2 with Control-IQ Automated Insulin Delivery System in Toddlers and Preschoolers

**DOI:** 10.1089/dia.2020.0507

**Published:** 2021-04-20

**Authors:** Laya Ekhlaspour, Melissa J. Schoelwer, Gregory P. Forlenza, Mark D. DeBoer, Lisa Norlander, Liana Hsu, Ryan Kingman, Emily Boranian, Cari Berget, Emma Emory, Bruce A. Buckingham, Marc D. Breton, R. Paul Wadwa

**Affiliations:** ^1^ Division of Endocrinology and Diabetes, Department of Pediatrics, Stanford University, Palo Alto, California, USA.; ^2^ Stanford Diabetes Research Center, Stanford, California, USA.; ^3^ Center for Diabetes Technology, University of Virginia, Charlottesville, Virginia, USA.; ^4^ Department of Pediatrics, University of Virginia, Charlottesville, Virginia, USA.; ^5^ Barbara Davis Center for Diabetes, University of Colorado Anschutz Medical Campus, Aurora, Colorado, USA.

**Keywords:** Type 1 diabetes, Closed loop, Artificial pancreas, Pediatrics

## Abstract

***Background:*** Glycemic control is particularly challenging for toddlers and preschoolers with type 1 diabetes (T1D), and data on the use of closed-loop systems in this age range are limited.

***Materials and Methods:*** We studied use of a modified investigational version of the Tandem t:slim X2 Control-IQ system in children aged 2 to 5 years during 48 h in an outpatient supervised hotel (SH) setting followed by 3 days of home use to examine the safety of this system in young children. Meals and snacks were not restricted and boluses were estimated per parents' usual routine. At least 30 min of daily exercise was required during the SH phase. All participants were remotely monitored by study staff while on closed-loop in addition to monitoring by at least one parent throughout the study.

***Results:*** Twelve participants diagnosed with T1D for at least 3 months with mean age 4.7 ± 1.0 years (range 2.0–5.8 years) and hemoglobin A1c of 7.3% ± 0.8% were enrolled at three sites. With use of Control-IQ, the percentage of participants meeting our prespecified goals of less than 6% time below 70 mg/dL and less than 40% time above 180 mg/dL increased from 33% to 83%. Control-IQ use significantly improved percent time in range (70–180 mg/dL) compared to baseline (71.3 ± 12.5 vs. 63.7 ± 15.1, *P* = 0.016). All participants completed the study with no adverse events.

***Conclusions:*** In this brief pilot study, use of the modified Control-IQ system was safe in 2–5-year-old children with T1D and improved glycemic control.

## Introduction

The glycemic targets for young children (age younger than 6 years) with type 1 diabetes (T1D) have traditionally been set higher than older children and adolescents primarily due to concerns regarding hypoglycemia. However, newer recommendations have stressed the importance of intensive management even for the youngest children with T1D and hemoglobin A1c [(HbA1c) goals <7.0%] are now the same across all pediatric ages.^1,2^ This shift was driven by recent data on the negative impact of hyperglycemia and glucose variability on the developing brain^3–5^ along with the observation that poor glycemic control does not protect against severe hypoglycemia.^6^ In addition, the availability of newer technologies, such as continuous glucose monitors (CGMs), has allowed caregivers to better identify and prevent hypoglycemia.^7,8^

Nevertheless, achieving a lower HbA1c in this age range remains incredibly challenging, and data from the T1D Exchange Clinic Network have shown that only 27% of young children with T1D attain even the former recommended target HbA1c of <7.5%.^9,10^ The difficulty in managing diabetes at this age is due to a number of factors, including increased insulin sensitivity, higher glycemic variability, the need for small amounts of insulin, erratic eating and activity patterns, inability to communicate symptoms of hypoglycemia and hyperglycemia, and parental fear of hypoglycemia.^11^ Rapid changes in growth and development also influence insulin sensitivity, resulting in a need for frequent dose adjustments.

Closed-loop automated insulin delivery systems, which combine an insulin pump with a CGM and a predictive algorithm that adjusts insulin delivery in real-time (“artificial pancreas”), have been shown to improve glycemic control in children, adolescents, and adults with T1D without increasing the risk of hypoglycemia.^12^ However, there are limited data on the use of these systems in toddlers and preschoolers^13–17^ and neither of the two Food and Drug Administration (FDA)-approved hybrid closed-loop systems are currently approved for use in this age range.

Therefore, in this study, we sought to evaluate the safety and efficacy of a modified version of the Tandem t:slim X2 Control-IQ system in young children with T1D, 2 to 5 years old, first under constant study team supervision and then for a brief period at home in their usual environment.

## Research Design and Methods

The Institutional Review Boards at the University of Virginia, the University of Colorado Anschutz Medical Campus, and Stanford University approved this study as well as the FDA (IDE G190205). They study was registered with clincialtrials.gov (NCT04084171). Written informed consent was obtained from a parent or guardian of every subject; written assent was not obtained due to age <6 years. Participants were considered for inclusion in the study if they were 2 to 5 years old at the time of the study, had been diagnosed with T1D for at least 3 months, were current users of an insulin pump and Dexcom CGM, and had a total daily insulin dose of at least 5 U. Participants were excluded from the study if they had a severe hypoglycemic event or diabetic ketoacidosis in the previous 3 months or if they used diluted insulin or any noninsulin glucose-lowering agent.

A medical history was obtained and a physical examination was performed at the screening visit along with a HbA1c and confirmation of CGM use for at least 11 out of the previous 14 days. Once eligibility criteria were confirmed, parents or guardians were trained on the study devices (Tandem t:slim X2™ insulin pump with Control-IQ™ and a Dexcom G6^®^ CGM) by a qualified trainer. Control-IQ as described in previous articles^18^ requires entry of a weight of at least 55 lbs (25 kg) and total daily insulin of at least 10 U/day. This study utilized a modified, investigational version of Control-IQ (Control-IQ Pro), which removed the weight variable and lowered the total daily insulin lower limit to 5 U/day. There was no modification to the algorithm itself, which is a model predictive control (MPC) developed at the University of Virginia and no change in the algorithm's functionality. Participants were also provided with a study glucometer (Accu-Chek^®^ Guide) and blood ketone meter (Abbott Precision Xtra^®^ Monitoring System). The study pump was programmed with the participant's usual insulin parameters and basal rates were adjusted, if necessary, to allow for the minimum programmable basal rate of 0.1 U/h. Optimization of parameters based on observed trends was allowed throughout the study as determined by a study physician. Following device training, participants used the study pump at home in open-loop (Control-IQ turned off, without Basal-IQ functionality) for 2 to 7 days before the outpatient supervised hotel (SH) admission to become familiar with the study pump.

Control-IQ was activated upon arrival to the SH setting, which was staffed at all times by a physician and other trained medical personnel. The parent or guardian of each participant was responsible for use of the system during the 48-h SH phase, including the timing and size of meal boluses, and the medical staff was available for assistance as needed. No restrictions were placed on the size of the meals or the frequency of snacks. Planned group activities occurred across all sites, including at least 30 min of exercise each day. Following the SH phase, each participant continued using the Control-IQ system at home for 72 h under parental supervision, and remote study staff monitoring. During the at home phase, participants were required to be with a parent or guardian who was trained on the system at all times. Parents completed the Technology Acceptance Questionnaire (scored on a 0–6 Likert scale)^19^ at the end of the study to determine if they trusted the system and if they felt it was easy to use and beneficial for managing their child's diabetes.

### Remote monitoring and safety protocols

Parents used Dexcom Share to remotely monitor CGM trends during the entirety of the study. The Dexcom Share application was programmed with the low alarm set at 70 mg/dL or higher and the high alarm programmed to no higher than 250 mg/dL. In addition, the study team remotely monitored participants' CGM trends while using Control-IQ during the SH phase and during the at home phase in real-time with alarms set as follows: <70 mg/dL for 15 min or >300 mg/dL for 60 min. The study staff contacted the family if either of these conditions were met to ensure that appropriate treatment was underway (directly during the SH admission or by phone during the at home portion of the study). The study team was also available by phone at all times during the at home portion of the study.

Blood ketone levels were checked upon admission and discharge of the SH phase. While the majority of treatment decisions were based on CGM readings, parents of participants were asked to perform self monitoring of blood glucose checks on arrival for the SH stay and any time the CGM reading was >300 mg/dL for greater than 60 min. Participants were treated with ∼16 g fast-acting carbohydrates for CGM readings <80 mg/dL during the day and <70 mg/dL overnight (or at higher glycemic thresholds per parent discretion). A repeat treatment was considered if CGM value was <80 mg/dL after ∼20 min. Hypoglycemic treatments could occur at any time per study physician request. Blood ketones were checked for a CGM reading >300 mg/dL for 2 h or >400 mg/dL at any point in time.

### Outcomes and statistical analysis

The prespecified primary outcome was the number of subjects with <6% time below 70 mg/dL and <40% time above 180 mg/dL as measured by CGM. Main comparison was baseline (2–7 days, median 4 days) versus Control-IQ use (5 days) using a related-sample McNemar change test with significance at 0.05. Additional secondary outcomes included percent time in range (TIR, 70–180 mg/dL), percent time spent in hypo- and hyperglycemia (<70 mg/dL, <60 mg/dL, <54 mg/dL, <50 mg/dL, >180 mg/dL, >250 mg/dL, >300 mg/dL), number of hypoglycemic events, time spent in closed-loop, and the number of participants meeting the CGM consensus target of >70% TIR with <4% below 70 mg/dL. CGM-based secondary outcomes were also compared to normative data obtained from 30 children with T1D (ages 6–12 years) managed with CGM and open-loop insulin pump therapy in France (baseline characteristics from interim analysis, NCT03739099). Comparisons between study phases (open-loop baseline [B]; closed-loop SH; closed-loop home [H], or H&SH combined as Control-IQ [CIQ]) were performed using two-sided paired Student's *t*-test for normally distributed outcomes or a related-sample Wilcoxon signed-rank test for skewed outcomes (e.g., percent time below 60 mg/dL) and related sample McNemar test for proportions (primary outcome and Consensus Goal). Outcomes are reported as mean ± standard deviation or median [interquartile range] unless otherwise indicated.

## Results

### Study participants

Twelve participants were enrolled across three sites (four at each site: Stanford University, University of Virginia, and Barbara Davis Center for Diabetes) and all subjects completed every phase of the study. Baseline demographic data are shown in Table 1. The average age was 4.7 ± 1.0 years with a range of 2.0 to 5.8 years old. The baseline HbA1C was 7.3% ± 0.8%. Two key inclusion criteria were use of an insulin pump in the past 3 months and use of Dexcom G6. All participants used either a sensor augmented pump or a closed-loop system before their enrollment visits. Three of the 12 participants (25%) were using a predictive low glucose suspend system before the study, and two (17%) were using a closed-loop system. The remaining seven participants were using sensor-augmented pump therapy.

**Table 1.** Baseline Participants Data

	Mean ± SD	Minimum–maximum
Gender	67% female	
Age (years)	4.7 ± 1.0	2–5.8
Diabetes duration (years)	2.08 ± 0.89	0.71–3.5
HbA1c (%)	7.3 ± 0.8	6.2–8.6
Weight (kg)	19.3 ± 3.1	13.2–24.9
Total insulin dose [U/(kg·day)]	0.76 ± 0.14	0.55–1.02
Total daily insulin (units)	14.6 ± 3.5	7.9–20.7

HbA1c, hemoglobin A1c; SD, standard deviation.

### Glycemic outcomes

CGM-based glycemic outcomes are outlined in Table 2. At baseline, 33% of participants achieved the primary outcome of less than 6% time in the hypoglycemic range (<70 mg/dL) combined with less than 40% time in the hyperglycemic range (>180 mg/dL) (Supplementary Fig. S1). Overall, 83% of participants met these criteria when using Control-IQ in either the SH or H phase, which is significantly more than the proportion of participants in the normative data cohort (83% vs. 65% *P* = 0.004) and at baseline (83% vs. 33% *P* = 0.031). TIR was 71.3% ± 12.5% on Control-IQ versus 61.7% ± 16.1% at baseline (*P* = 0.016). Control-IQ had the greatest impact on decreasing time in hyperglycemia >180 mg/dL (34.1% ± 17.3% at baseline vs. 25.7% ± 12.1% on Control-IQ, *P* = 0.042). This reduction was more significant overnight (38.9% ± 21.1% vs. 22.4% ± 16%). The percent time in hypoglycemia (<70 mg/dL) was similar on Control-IQ compared to baseline (3.2 [1.5%–4.6%] on Control-IQ vs. 3.7 [1.5%–6.4%] at baseline, *P* = 0.182). However, time <60 mg/dL with Control-IQ was half of the amount at baseline, but the difference was not significant (0.7 [0.5%–1.4%] vs. 1.6 [0.3%–2.8%], *P* = 0.110).

**Table 2.** Glycemic Outcomes (Baseline vs. Control-IQ)

Overall			
	Baseline	CIQ	P
Percent with less than 6% time below 70 mg/dL and less than 40% time above 180 mg/dL	33%	83%	0.031
CGM consensus goal	8%	58%	0.031
% time below 50 mg/dL	0.2 (0%–0.9%)	0.2 (0.1%–0.3%)	0.575
% time below 54 mg/dL	0.6 (0.1%–1.6%)	0.3 (0.2%–0.7%)	0.424
% time below 60 mg/dL	1.6 (0.3%–2.8%)	0.7 (0.5%–1.4%)	0.110
% time below 70 mg/dL	3.7 (1.5%–6.4%)	3.2 (1.5%–4.6%)	0.182
% time between 70 and 140 mg/dL	41.8 ± 15.9	51.5 ± 12.8	0.062
% time between 70 and 180 mg/dL	61.7 ± 16.1	71.3 ± 12.5	0.016
% time above 180 mg/dL	34.1 ± 17.3	25.7 ± 12.1	0.042
% time above 250 mg/dL	9.7 (6.1%–19.5%)	4.2 (2.3%–9%)	0.024
% time above 300 mg/dL	3.4 (0.6%–5.4%)	0.6 (0.1%–1.6%)	0.013
average CGM (mg/dL)	161.1 ± 28.1	147.1 ± 17.7	0.054
Glucose variability (CV, %)	39.1 ± 5.9	36.5 ± 3.7	0.121
Total insulin (U/day)	13.1 ± 3.8	13.6 ± 4	0.155
Total CHO (g/day)	106.5 ± 31.4	128.3 ± 40.8	0.019
percent time in closed loop (%)	—	96.6 (93.6%–98.9%)	
*Daytime (7 am–11 pm)*			
% time below 50 mg/dL	0 (0%–0.9%)	0.1 (0%–0.4%)	0.594
% time below 54 mg/dL	0.3 (0.1%–1.4%)	0.3 (0.1%–1%)	0.859
% time below 60 mg/dL	0.7 (0.4%–3.6%)	0.9 (0.5%–2.1%)	0.286
% time below 70 mg/dL	3.6 (1.5%–7.2%)	3.7 (1.9%–5.2%)	0.530
% time between 70 and 140 mg/dL	42.8 ± 15	49.6 ± 12.5	0.115
% time between 70 and 180 mg/dL	63.7 ± 15.1	69 ± 12.3	0.121
% time above 180 mg/dL	31.5 ± 16.5	27.3 ± 12.3	0.241
% time above 250 mg/dL	8.6 (1.7%–15%)	4.3 (2.2%–8.4%)	0.164
% time above 300 mg/dL	2.1 (0%–5.5%)	0.4 (0%–2.3%)	0.028
Average CGM (mg/dL)	157.1 ± 28.4	148.2 ± 18.4	0.173
Glucose variability (CV, %)	38.7 ± 6.3	37.2 ± 4.2	0.370
Total insulin (U/day)	10.6 ± 3	11.2 ± 3.3	0.115
percent time in closed loop (%)	—	96 (92.7%–98.6%)	—
*Overnight (11 pm–7 am)*			
% time below 50 mg/dL	0 (0%–0%)	0 (0%–0.7%)	0.173
% time below 54 mg/dL	0 (0%–1%)	0 (0%–0.9%)	0.735
% time below 60 mg/dL	0.7 (0%–2.2%)	0 (0%–1.3%)	0.260
% time below 70 mg/dL	2.1 (1.4%–4.9%)	1.3 (0.4%–2.3%)	0.239
% time between 70 and 140 mg/dL	39.8 ± 19.9	55.3 ± 19.2	0.043
% time between 70 and 180 mg/dL	57.8 ± 19.7	75.9 ± 17	0.004
% time above 180 mg/dL	38.9 ± 21.1	22.4 ± 16	0.010
% time above 250 mg/dL	11.7 (7.6%–21.4%)	3.3 (2%–7.2%)	0.027
% time above 300 mg/dL	2.1 (0%–6.2%)	0 (0%–0.4%)	0.093
average CGM (mg/dL)	168.3 ± 33.8	144.7 ± 22.1	0.028
Glucose variability (CV, %)	37.2 ± 5.8	32.7 ± 5.9	0.060
Total insulin (U/night)	2.5 ± 1	2.4 ± 0.8	0.493
percent time in closed loop (%)	—	99.7 (90.5%–100%)	—

CGM, continuous glucose monitor; CHO, carbohydrates; CV, coefficient of variation.

Nonpowered explorations by study phase (B vs. SH vs. H) are reported in Table 3. TIR was highest during SH (76.5% ± 15.5%, *P* < 0.001 vs. B) and hypoglycemia was lowest during Control-IQ use at home (SH: 5 [3.3%–5.7%] vs. H: 1.5 [0.4%–2.9%]; *P* = 0.002). During the SH, hyperglycemia >180 mg/dL was reduced by half compared to baseline (*P* = 0.003), which equates to four fewer hours >180 mg/dL per day. The reduction in hyperglycemia with Control-IQ was most pronounced overnight (B: 38.9% ± 21.1% vs. SH: 18.9% ± 19.1%; *P* = 0.003, H: 24.4% ± 18.7%; *P* = 0.042).

**Table 3.** Glycemic Outcomes by Study Phases (Baseline, Supervised Hotel and Home)

Overall						
	Mean ± SD or median (quartiles)			P		
Glycemic outcomes	Baseline (B)	SH	Home (H)	SH vs. B	H vs. B	H vs. SH
Percent <50 mg/dL (%)	0.2 (0%–0.9%)	0.2 (0.1%–0.4%)	0 (0%–0.3%)	0.922	0.148	0.055
Percent <54 mg/dL (%)	0.6 (0.1%–1.6%)	0.6 (0.4%–0.8%)	0.1 (0%–0.7%)	0.966	0.074	**0.024**
Percent <60 mg/dL (%)	1.6 (0.3%–2.8%)	1.4 (1%–1.8%)	0.2 (0%–1.2%)	0.898	**0.042**	**0.024**
Percent <70 mg/dL (%)	3.7 (1.5%–6.4%)	5 (3.3%–5.7%)	1.5 (0.4%–2.9%)	0.233	0.052	**0.002**
Percent 70–140 mg/dL (%)	41.8 ± 15.9	58.8 ± 17	46.8 ± 12.5	**0.007**	0.319	**0.006**
Percent 70–180 mg/dL (%)	61.7 ± 16.1	76.5 ± 15.5	68 ± 12.4	**0.001**	0.125	**0.021**
Percent >180 mg/dL (%)	34.1 ± 17.3	18.6 ± 14.9	30.1 ± 12.2	**0.001**	0.343	**0.003**
percent >250 mg/dL (%)	12.1 ± 9.4	3.6 ± 5.1	8.8 ± 8.7	**0.003**	0.153	**0.004**
Percent >300 mg/dL (%)	3.4 (0.6%–5.4%)	0 (0%–0%)	0.9 (0%–2.3%)	**0.004**	**0.027**	0.074
Mean CGM (mg/dL)	161.1 ± 28.1	134 ± 20.5	155.2 ± 18.3	**0.001**	0.42	**<0.001**
Glucose variability (CV, %)	39.1 ± 5.9	34.6 ± 4.6	35.5 ± 4.2	**0.0498**	**0.047**	0.53
Total insulin [U/(kg·day)]	0.67 ± 0.15	0.63 ± 0.18	0.74 ± 0.15	0.232	**<0.001**	**0.009**
Total CHO (g/day)	106.5 ± 31.4	123.6 ± 53.8	129.8 ± 40.5	0.297	**0.002**	0.447
Number of hypoglycemic events	2.13 ± 1.36	2.76 ± 0.85	1.19 ± 1.06	0.134	**<0.001**	0.100
Percent time in closed loop (%)	—	98.7 (93.4%–99.5%)	97.4 (95%–98.7%)	—	—	0.614
*Daytime (7 am–11 pm)*						
	*Baseline (B)*	*SH*	*Home (H)*	*SH vs. B*	*H vs. B*	*H vs. SH*
Percent <50 mg/dL (%)	0 (0%–0.9%)	0.3 (0%–0.4%)	0 (0%–0.4%)	0.375	0.820	0.469
Percent <54 mg/dL (%)	0.3 (0.1%–1.4%)	0.7 (0%–1.1%)	0.1 (0%–1%)	0.898	0.098	0.160
Percent <60 mg/dL (%)	0.7 (0.4%–3.6%)	1.7 (0.6%–2.4%)	0.3 (0%–1.8%)	0.831	0.054	0.064
Percent <70 mg/dL (%)	3.6 (1.5%–7.2%)	6.2 (4.3%–8.4%)	1.9 (0.6%–3.5%)	0.092	0.077	**0.001**
Percent 70–140 mg/dL (%)	42.8 ± 15	58.8 ± 17.1	44.1 ± 11.9	**0.006**	0.750	**0.001**
Percent 70–180 mg/dL (%)	63.7 ± 15.1	75.5 ± 14.9	65.1 ± 12.9	**0.007**	0.667	**0.007**
Percent >180 mg/dL (%)	31.5 ± 16.5	18.4 ± 14.9	32.6 ± 13	**0.004**	0.788	**0.001**
Percent >250 mg/dL (%)	10 ± 9.3	3.8 ± 5.3	9 ± 8.6	**0.012**	0.663	**0.002**
Percent >300 mg/dL (%)	2.1 (0%–5.5%)	0 (0%–0.1%)	0.6 (0%–3.3%)	**0.008**	**<0.001**	0.078
Mean CGM (mg/dL)	157.1 ± 28.4	132.7 ± 21.7	157.2 ± 18.9	**0.002**	0.984	**<0.001**
Glucose variability (CV, %)	38.7 ± 6.3	35.7 ± 4.7	35.7 ± 4.8	0.188	0.100	0.978
Total Insulin [U/(kg·day)]	0.55 ± 0.12	0.51 ± 0.15	0.61 ± 0.13	0.317	**<0.001**	**0.013**
Total CHO (g/day)	103.5 ± 29.6	120.8 ± 53.8	126.6 ± 40.5	0.308	**0.006**	0.546
Number of hypoglycemic events	1.71 ± 1.18	2.42 ± 0.9	1.03 ± 0.93	0.084	**0.001**	0.122
*Night (11 pm–7 am)*						
	*Baseline (B)*	*SH*	*Home (H)*	*SH vs. B*	*H vs. B*	*H vs. SH*
Percent <50 mg/dL (%)	0 (0%–0%)	0 (0%–1.2%)	0 (0%–0%)	0.500	0.563	0.125
Percent <54 mg/dL (%)	0 (0%–1%)	0 (0%–1.3%)	0 (0%–0%)	0.594	0.375	0.063
Percent <60 mg/dL (%)	0.7 (0%–2.2%)	0 (0%–2.4%	0 (0%–0%	0.945	0.098	0.063
Percent <70 mg/dL (%)	2.1 (1.4%–4.9%	1.8 (0%–4.8%	0 (0%–1.3%	0.413	0.105	0.371
Percent 70–140 mg/dL (%)	39.8 ± 19.9	58.8 ± 21.9	53.1 ± 24.5	**0.028**	0.113	0.476
Percent 70–180 mg/dL (%)	57.8 ± 19.7	78.6 ± 21.4	74.5 ± 18.8	**0.003**	**0.019**	0.531
Percent >180 mg/dL (%)	38.9 ± 21.1	18.9 ± 19	24.4 ± 18.7	**0.003**	**0.042**	0.376
Percent >250 mg/dL (%)	15.9 ± 14	3.2 ± 5.1	8.3 ± 10.7	**0.007**	0.100	**0.044**
Percent >300 mg/dL (%)	2.1 (0%–6.2%	0 (0%–0%	0 (0%–0.1%	0.078	**<0.001**	0.625
Mean CGM (mg/dL)	168.3 ± 33.8	136.3 ± 23.1	150.4 ± 27.2	**0.008**	0.102	0.087
Glucose variability (CV, %)	37.2 ± 5.8	29.6 ± 8	30.8 ± 7.3	**0.031**	**0.024**	0.619
Total Insulin [U/(kg·day)]	0.13 ± 0.04	0.12 ± 0.05	0.12 ± 0.03	0.612	0.782	0.879
Total CHO (g/day)	3 ± 6.8	2.9 ± 6.9	3.2 ± 5.2	0.878	0.311	0.394
Number of hypoglycemic events	0.48 ± 0.36	0.39 ± 0.32	0.19 ± 0.32	0.615	0.200	0.175

*P* values in bold are statistically significant (*P* < 0.05)

SH, outpatient supervised hotel.

**FIG. 1.**
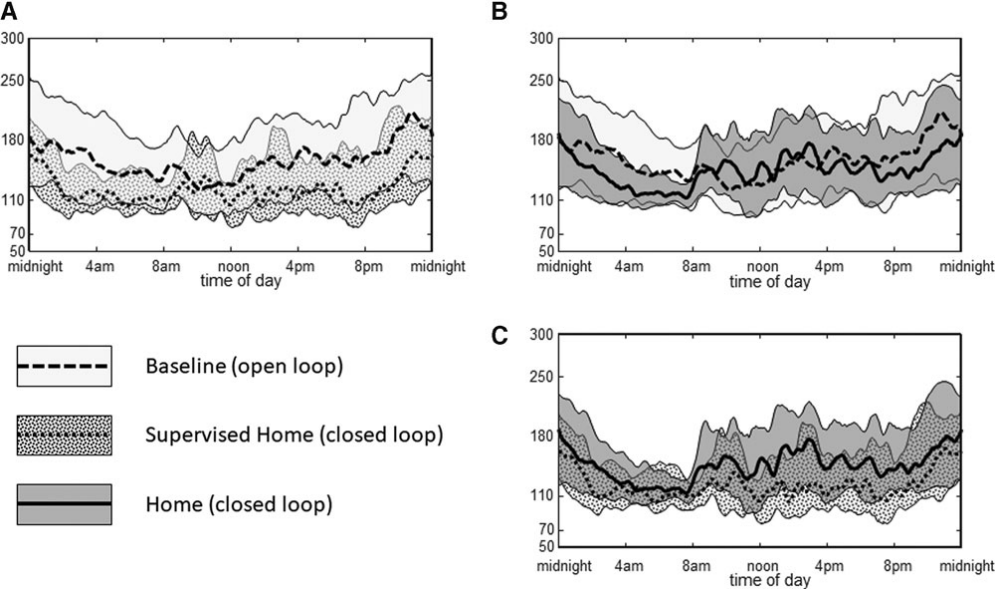
Comparison of Modal Day Graphs throughout the study **(A)**. Baseline versus Supervised Home phase **(B)**. Baseline Versus Home Phase **(C)**. Supervised Home Versus Home Phase.

Sensor glucose (SG) profiles during each phase of the study are provided in Figure 1. Compared to baseline, average glucose values were lower throughout the day without increased exposure to hypoglycemia with use of Control-IQ (Fig. 1a, b). Mean SG was also lower during the daytime SH phase compared to the mean daytime SG during the baseline, open-loop phase (132.7 ± 21.7 mg/dL vs. 157.1 ± 28.4 mg/dL, *P* = 0.002). The improved glycemic control was more pronounced overnight compared to daytime, particularly from midnight to 8 AM, when the mean SG decreased from 168.1 ± 33.7 mg/dL to 136.1 ± 23.2 mg/dL (*P* < 0.008).

The total daily dose of insulin was not significantly different between baseline and SH, but was higher during the home phase of the study [B: 0.67 ± 0.15 vs. SH: 0.63 ± 0.18 U/(kg·day); *P* = 0.232, H: 0.74. ± 0.15, *P* < 0.001]. Total insulin use was not significantly different between phases during the overnight period.

### Percentage time in closed-loop

During the supervised phase, participants remained in closed-loop 98.4% of the time. This did not decrease significantly during the at-home phase where closed-loop was in use 97.5% of the time.

### Human factors

**FIG. 2.**
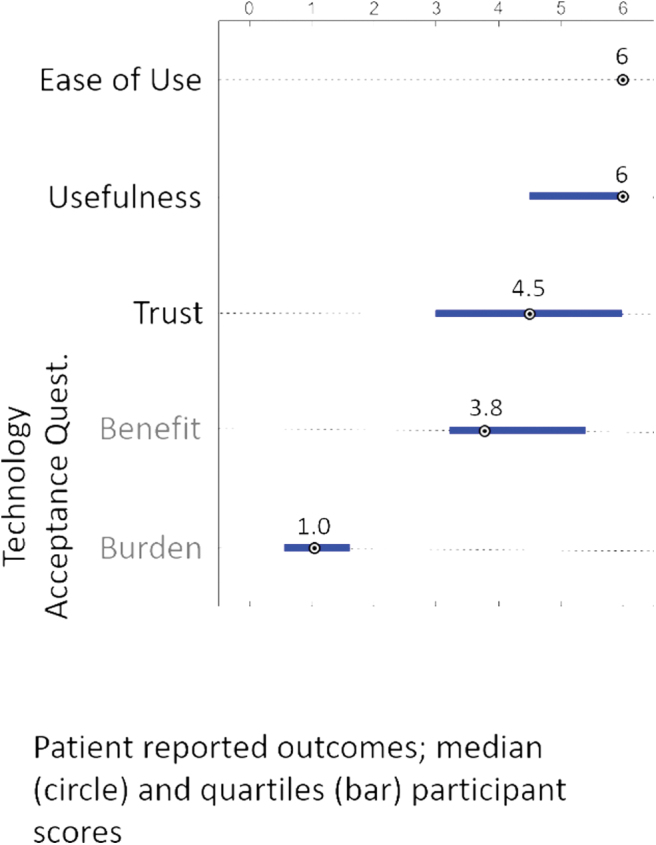
Technology Acceptance Questionnaire Results. Color images are available online.

Results from the Technology Acceptance Questionnaire revealed that the parents had favorable subjective responses to the system, with the high scores on ease of use and usefulness with minimal burden reported (Fig. 2).

### Safety outcomes and adverse events

There were no severe hypoglycemia events and no episodes of diabetic ketoacidosis. All participants completed the study without meeting early discontinuation criteria. There was an average of 2.8 hypoglycemic events per day during the supervised phase and 1.2 events/day during use at home, compared to 2.1 events/day at baseline; with 0.5 events/night, 0.4 events/night, and 0.2 events/night for B, SH, and H use, respectively.

## Discussion

The data from this pilot study indicate that this modified version of the commercial Tandem t:slim X2 Control-IQ system appears safe for use in young children 2–5 years old. In addition, Control-IQ improved glycemic control by increasing TIR without increasing hypoglycemia. This improvement in glycemic outcomes occurred despite variability in the participants' activity level and meal intake during the SH phase. Rates of clinical hypoglycemia (<54 mg/dL) were significantly lower during the home use of Control-IQ than baseline and similar during the increased challenges of SH, including increased physical activity in a semi-camp setting as well as being away from routine eating habits, in toddlers participating in the study. Compared to the baseline, all overnight outcomes were improved with the use of Control-IQ and daytime hyperglycemia (>300 mg/dL) significantly decreased.

Furthermore, the Control-IQ system was found to be highly reliable in this study. The children were in closed-loop for over 97% of the 5 days of use, which is similar to data reported in older populations in studies of longer duration.^18,20^ Although studies of longer duration are needed to confirm these findings, a high degree of reliability of the system may lead to a reduction in diabetes burden, a key goal in managing individuals with diabetes, particularly for toddlers and their parents. The system also scored high for usability by the parents as measured by their responses to the Technology Acceptance Questionnaire.

Numerous randomized trials have assessed the current Control-IQ algorithm in older children, adolescents, and adults, noting comparable improvements in glycemic control across a number of settings.^14,18,20^ The FDA recently approved the Tandem Control-IQ system for use in people with T1D aged 6 years and older. We previously reported the results of the same algorithm during home use for 3 days in children 6–12 years old following a ski camp. During that trial, Control-IQ similarly resulted in an improvement in TIR (71.0% vs. 52.8%) without increasing the time <70 mg/dL.

Brown et al. investigated the efficacy of Control-IQ during home use in a randomized controlled trial with 168 participants (14–71 years old) for 6 months. They reported an increase in TIR from a baseline of 61% ± 17% to 71% ± 12% in the closed-loop group and no change in TIR in the control group, using sensor augmented pump therapy (mean adjusted difference, 11 percentage points; 95% confidence interval [CI], 9–14; *P* < 0.001).^18^ A subsequent 16-week pivotal trial conducted in 101 children, aged 6–13 years, reported similar difference in TIR in participants using Control-IQ compared to the control group.^21^ Our current results indicate that similar improvement in glycemic control was realized in toddlers and preschoolers on this modified version of Control-IQ.

To date, only a few small studies have evaluated closed-loop systems in children younger than the age of 6 years. One randomized controlled crossover trial compared closed-loop with standard open-loop insulin pump therapy only from 10 pm to 12 pm on two consecutive days at an inpatient clinical research center.^13^ Results from this study indicate a trend toward a higher overnight TIR (70–200 mg/dL) in the closed-loop group, although this was not significant.^13^

Two studies by Elleri et al. studied a closed-loop system in a total of 35 young children (age range 1–7 years) with either diluted insulin aspart or standard U100 insulin aspart and found similar glycemic outcomes between both insulin groups and reported no adverse events.^15,17^

A retrospective analysis conducted by Salehi et al. of the Medtronic 670G hybrid closed-loop system in children younger than 7 years old reported significantly increased TIR (42.8%, to 56.2%, *P* < 0.001) and lower average SG (200 mg/dL vs. 176 mg/dL *P* < 0.001). However, the percentage of hypoglycemia increased from 1.3% to 2.4% (*P* = 0.04).^16^

Recently, Buckingham et al. studied the safety and performance of the Omnipod^®^ hybrid closed-loop personalized MPC algorithm in children with T1D aged 2–5.9 years in a SH setting over 48–72 h. TIR increased by 32% overall and 47% overnight. There was also an ∼2-fold reduction in percent time <70 mg/dL.^22^ Notably, in all of these studies of closed-loop control in young children, there were no significant serious adverse events reported among users.

There are a number of limitations to our study, including the short duration of Control-IQ use with inadequate time to optimize pump settings during a short baseline period. In addition, because this was the first outpatient study utilizing Control-IQ in toddlers and preschoolers, a high degree of physician oversight was provided, including remote monitoring for the entirety of closed-loop use as well as parental monitoring. This high level of oversight may have biased the results toward better control than would be expected in the usual outpatient setting. Also, this study was not randomized and most participants in this study had good glycemic control at baseline with enrollment HbA1c values <7.5% on average, which is lower than those in most young children with T1D. All of our participants were on sensor-augmented pumps at enrollment, while 25% were using a system with predictive low glucose suspend, and 17% were using a do-it-yourself closed-loop system (LooP). Due to the parents' high interest in using a closed-loop system, extending the results to a wider spectrum of children with diabetes in this age range should be done cautiously, and a larger, longer outpatient trial is warranted. Since the previous clinical trials testing the efficacy of Control-IQ have been inclusive of a wide range of HbA1c levels,^18,21^ we expect toddlers with higher HbA1cs will similarly benefit from the use of this closed-loop system.

In conclusion, the modified investigational version of the Tandem t:slim X2 with Control-IQ System significantly improved glycemic control without an increase in hypoglycemia in toddlers and preschool-aged children with T1D. Larger clinical trials of longer duration are required to expand on experience with this system for this age group.

## Supplementary Material

Supplemental data
