# Supplementary material for: Safety and Performance of the Tandem t:slim X2 with Control-IQ Automated Insulin Delivery System in Toddlers and Preschoolers
Source: Diabetes Technol Ther. 2021 Apr 20;23(5):384–91. doi: 10.1089/dia.2020.0507 (PMC8080923; doi:10.1089/dia.2020.0507)

Figure 1S. Percentage of participants meeting goal of less than 6% time below 70 mg/dL and less than 40% time above 180 mg/dL


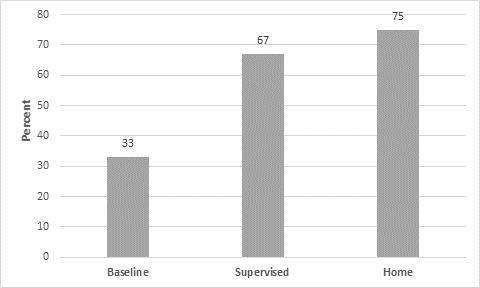

Supplement: Supplemental data [file Supp_FigS1.docx]
